# Deconstructing risk: Separable encoding of variance and skewness in the brain

**DOI:** 10.1016/j.neuroimage.2011.06.087

**Published:** 2011-10-15

**Authors:** Mkael Symmonds, Nicholas D. Wright, Dominik R. Bach, Raymond J. Dolan

**Affiliations:** Wellcome Trust Centre for Neuroimaging, Institute of Neurology, University College London, 12 Queen Square, London WC1N 3BG, UK

**Keywords:** Decision making, fMRI, Risk, Variance, Skewness

## Abstract

Risky choice entails a need to appraise all possible outcomes and integrate this information with individual risk preference. Risk is frequently quantified solely by statistical variance of outcomes, but here we provide evidence that individuals’ choice behaviour is sensitive to both dispersion (variance) and asymmetry (skewness) of outcomes. Using a novel behavioural paradigm in humans, we independently manipulated these ‘summary statistics’ while scanning subjects with fMRI. We show that a behavioural sensitivity to variance and skewness is mirrored in neuroanatomically dissociable representations of these quantities, with parietal cortex showing sensitivity to the former and prefrontal cortex and ventral striatum to the latter. Furthermore, integration of these objective risk metrics with subjective risk preference is expressed in a subject-specific coupling between neural activity and choice behaviour in anterior insula. Our findings show that risk is neither monolithic from a behavioural nor neural perspective and its decomposition is evident both in distinct behavioural preferences and in segregated underlying brain representations.

## Highlights

► Behavioural influence of variance and skewness (asymmetry) of risky decisions. ► Posterior parietal cortex is sensitive to variance. ► Distributed skewness representation in striatum, prefrontal and insular cortex. ► Integration of decision statistics and individual risk preferences in anterior insula.

## Introduction

When foraging animals, or humans in a modern economy, make a decision they must evaluate potential outcomes of a choice and the chance of each outcome occurring. For example, imagine purchasing house A, whose market value 5 years hence could be either £115 k (with a 70% chance), £100 k (15% chance), or £30 k (15% chance). How much is this house worth? Alternatively, house B that might be worth £170 k (with a 15% chance), 100 k (15% chance), or £85 k (70% chance). Which house is preferred? Considering only the expected value (i.e. mean) we should be indifferent because both are worth exactly £100 k. However, the price one is willing to pay may also depend upon individuals’ taste for the risk involved with each option (their “risk preference”).

Risk is psychologically multi-faceted and, in our example, could relate to the spread of outcomes (variance) or asymmetry between better or worse than average outcomes (skewness). A taste for variance dictates both houses are equally valued (both sets of outcomes have equal variance). Preferring one of the houses suggests an additional predilection for negatively skewed (house A—small chance of a poor outcome), or positively skewed (house B—small chance of a good outcome) distributions. Here, we aimed to identify neural mechanisms that evaluate these different aspects of risk and determine how they are integrated with individuals’ preferences for each.

There are two dominant theories of risk evaluation. In microeconomics, Expected Utility Theory (Von Neumann and Morgenstern, 1944) proposes that decision-makers’ subjective values for each possible outcome are determined by an implicit utility function, with ‘utilities’ weighted by outcome probabilities and risk preference emerging as a by-product of this framework (Pratt, 1964). Alternative theories in finance (Markowitz, 1952), psychology (Coombs and Huang, 1970), and ecology (Stephens, 1981) propose that outcome distributions are decomposed into “summary statistics” (e.g. mean, variance, skewness), with risk preference directly generated by preference for each component. Observations of behaviour alone cannot distinguish Expected Utility from summary statistic models since both theories make identical choice predictions, as any utility function can be approximated by preferences for summary statistics using a polynomial expansion (Scott and Horvath, 1980). Critically, choice-generated neural data can provide important evidence in adjudicating between behavioural models. Thus, we used model-based fMRI analysis (O'Doherty et al., 2007) to determine whether the brain encodes the summary statistics (variance and skewness) of a decision.

Previous studies suggest involvement of dorsomedial prefrontal cortex (DMPFC), anterior insula, posterior parietal cortex (PPC), and ventral striatum in risk-related decision making (Mohr et al., 2010a). Several of these studies focus on variance (Christopoulos et al., 2009; Preuschoff et al., 2006; Tobler et al., 2007, 2009), but this measure ignores the possible influence of positive or negative skewness. We hypothesised that variance and positive/negative skewness would be distinctly represented within these regions. As these summary statistics need to be integrated with an individuals’ taste for risk, we were also interested in identifying where in the brain objective (task-based) and subjective (disposition to risk) variables are assimilated prior to a decision.

## Materials and methods

The study was approved by the Institute of Neurology (University College London) Ethics Committee. 24 subjects (mean age: 24; age range: 19–34; male: 12) participated in the experiment. 1 (female) subject was excluded because they used a fixed strategy (always chose sure amount), hence behavioural preferences could not be estimated. We provided a 5-minute practice tutorial to demonstrate the paradigm. Stimuli were presented and responses recorded using Cogent presentation software (Wellcome Trust Centre for Neuroimaging, London) written in MATLAB (version 6.5, MathWork, Natick, MA). Imaging data were analysed using Statistical Parametic Mapping software (SPM8; Wellcome Trust Centre for Neuroimaging, UK). Visual cues were projected onto a screen, visible via an angled mirror mounted on the MRI head coil. Choices were indicated by pressing a button box with the right index finger.

### Task

**Fig. 1:**
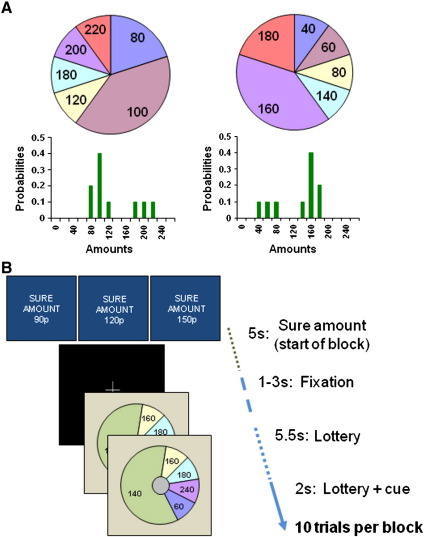
Experimental Paradigm. A. We represented gambles on-screen as pie-charts. The pie chart was divided into different segments showing possible outcomes from the lottery. The numbers written in each segment showed the monetary value of each outcome in pence (sterling) and the angle subtended by each segment indicated the probability of each outcome occurring. A positively skewed gamble (left) has a small chance of a better than average outcome (the tail of the distribution is to the right). Conversely, a negatively skewed gamble (right) has a small chance of a worse than average outcome (the tail is to the left). Both example gambles have identical variance and expected value. B. The task consisted of trials, grouped into experimental blocks of ten. For each trial, a pie chart was shown and after 5.5 s, a cue to respond appeared on screen (for 2 s). Subjects indicated by a button press while the cue was on-screen if they wanted to gamble on the lottery, or alternatively select a fixed, sure amount of money. To commence a block, the sure amount was written on the screen (3 levels—90p, 120p, 150p). At the end of each block, one trial from the block was randomly selected and played out for real. If subjects had elected to gamble, we resolved the lottery by an on-screen graphic of a red ball spinning around the outside of the pie which stopped at a randomly selected position. 180 trials were presented in total (60 stimuli at each of 3 sure levels).

To dissociate different components of risk, in terms of dispersion (variance) and asymmetry of outcomes (skewness), we designed a novel decision-making task that controlled the distribution of outcomes, and ensured that variance and skewness of a set of lotteries were manipulated independently by design (Fig. 1A, Supplementary Fig. 1). Hence, as variance and skewness of gambles were orthogonal factors, we could test whether neural activity evoked by variance could be distinguished from that evoked by skewness. Participants were required to choose between taking a ‘sure’ (fixed) amount of money or electing to ‘gamble’ (choosing to play a lottery with a number of potential outcomes). Gambles were represented as pie-charts, where variance and skewness of outcomes varied over a range, with expected value kept constant (Fig. 1B). We predicted distinct preferences for both variance and skewness (possibly with different preferences for positive versus negative skewness).

### Independent manipulation of variance and skewness

We constructed a stimulus set of 60 lotteries where variance and skewness were independent and varied over a range (Supplementary Fig. 1, Supplementary Table 1). For every level of variance (10 levels), we independently varied skewness (6 levels, 3 positively skewed, 3 negatively skewed). Expected value of the lotteries was kept constant (between £1.26 and £1.34), while variance ranged from 0.1 to 0.7 £^2^, and (standardised) skewness ranged from − 1 to 1. Stimuli were constrained to have between 3 and 9 outcomes (segments of the pie chart), with outcome probabilities varying in minimum 0.1 increments between 0 and 1 to mitigate against probability distortion effects at small probabilities. These restrictions allow the generation of a space of possible lotteries varying in skewness and variance. We pre-specified our desired levels of variance and skewness and selected lotteries to give as orthogonal a stimulus set as possible. We also resampled our set of lotteries to ensure variance and skewness were decorrelated from the number of segments in each presented pie chart gamble (variance r^2^ = 0.01; skewness r^2^ = 0.0004). Where 2 lotteries were equidistant from our desired array of points, we selected a lottery at random. Using multiple outcomes is critical, as for binary gambles, it is impossible to independently manipulate statistical moments across a range of values.

To commence a block, the sure amount was written on the screen (3 levels—90p, 120p, 150p). At the end of each block (every 10 trials), one trial from the block was randomly selected and played out for real. If subjects had elected to gamble, we resolved the lottery by an on-screen graphic of a red ball spinning around the outside of the pie until it stopped at a randomly selected position. This procedure was also shown in the practice, to demonstrate the idea that the size of each segment of the pie chart represented the chance of that outcome occurring. Resolving one trial per block helped maintain subjects’ task engagement during and thus maximise sensitivity to detect evoked responses to the experimentally-manipulated stimulus dimensions. Importantly, we do not expect any shifts in individual behavioural preferences to change the evoked response to the objective features of the gamble stimuli themselves. In addition, any changes in behaviour can only count against (i.e. reduce the sensitivity of) an analysis of correlations between trial-by-trial choice, individual preference, and neural activity. 180 trials were presented in total (60 stimuli for each of the 3 sure levels). Monetary earnings ranged between £16.10 and £24.30 (mean £19.40).

### Behavioural modelling

For a given lottery with N potential outcomes *m*_*1*_*, m*_*2*_*,… m*_*N*_, with probabilities *p = p*_*1*_*, p*_*2*_, …p_N_, we define the statistical moments (expected value (EV), variance (Var), standardised skewness (Skw)) of the outcome distribution as follows:

(1) $$EV=\sum_{n=1}^{N}m_{n}p_{n}$$

(2) $$Var=\sum_{n=1}^{N}{\left(m_{n}-EV\right)}^{2}p_{n}$$

(3) $$Skw=\frac{\sum_{n=1}^{N}{\left(m_{n}-EV\right)}^{3}p_{n}}{Var^{3/2}}$$

We analysed choice data by fitting a linear mean-variance-skewness model (***MVS***) where individuals are allowed to express different preferences for variance and skewness, and compared this to a set of reduced models and a standard power utility model commonly used to model standard expected utility (Camerer, 2003). The reduced models included a model based on mean difference (**M**) alone (where subjects only take account of the difference between the sure amount and the expected value of the gamble in selecting actions), a mean-variance model (**MV**), and a mean-skewness (**MS**) model. We then define the subjective value, or utility (U) of each lottery for our models:Mean model (**M**):

(4) U=EV

Mean-variance model (**MV**):

(5) U=EV+ρVar

Mean-skewness model (**MS**):

(6) U=EV+λSkw

Mean-variance-skewness model (**MVS**):

(7) U=EV+ρVar+λSkw

ρ reflects preference for variance, λ indicates preference for positive versus negative skewness.Expected utility model (**EUT**):

(8) $$U=\sum_{n=0}^{N}\frac{m_{n}{}^{1-\kappa }\;p_{n}}{1-\kappa }$$

κ reflects the concavity of the utility (power) function, hence the degree of risk-aversion.

We also tested a further set of models, where subjects were allowed to express a preference separately for positive and negatively skewed gambles. These models are specified as:Mean-variance-positive skewness (**MVpS**):

(9) $$U=EV+\rho Var+\lambda_{p}Skw^{+}$$

Mean-variance-negative skewness (**MVnS**):

(10) $$U=EV+\rho Var+\lambda_{n}Skw^{-}$$

Mean-positive skewness-negative skewness model (**MpSnS**):

(11) $$U=EV+\lambda_{p}Skw^{+}+\lambda_{n}Skw^{-}$$

Mean-variance-positive skewness-negative skewness model (**MVpSnS**):

(12) $$U=EV+\rho Var+\lambda_{p}Skw^{+}+\lambda_{n}Skw^{-}$$

Where Skw^+^ indictates Skw ≥ 0 and Skw^-^ indicates Skw < 0, and λ_p_ and λ_n_ reflect preferences for positive and negative skewness respectively.

We also tested a probability weighting (**PW**) model, where probabilities are transformed according to a one-parameter probability weighting function (Prelec, 1998):

(13) $$U=\sum_{n=0}^{N}\frac{m_{n}{}^{1-\kappa }\;g(p_{n})}{1-\kappa }\;\text{where}\;g\left(p_{n}\right)=e^{-{\left(-\ln p_{n}\right)}^{\alpha }}\;$$

We finally tested a cumulative prospect theory (CPT) model (Tversky and Kahneman, 1992). For a given lottery with N potential outcomes, we redefine outcomes relative to a reference point *R*, such that the outcomes are *m*_*-T*_*, m*_*-T + 1*_*,m*_*-T + 2*_*…, R,… m*_*N − 2,*_ *m*_*N − 1,*_ *m*_*N*_, with probabilities *p = p*_*−T*_*, p*_*−T + 1*_*,p*_*−T + 2*_*…, p*_*R*_*,… p*_*N − 2,*_ *p*_*N − 1,*_ *p*_*N*_*.* Overall utility *U* = *U*^−^ + *U*^*R*^ + *U*^+^, is given as:For *m > R:*

(15) $$U^{+}=g\left(p_{N}\right)u\left(m_{N}\right)+\sum_{k=1}^{N}\left[g\left(\sum_{j=0}^{k}p_{N-j}\right)-g\left(\sum_{j=0}^{k-1}p_{N-j}\right)\right]u\left(m_{N-k}\right)$$

For *m < R:*

(16) $$U^{-}=g\left(p_{-T}\right)u\left(m_{-T}\right)+\sum_{k=1}^{T}\left[g\left(\sum_{j=0}^{k}p_{-T+j}\right)-g\left(\sum_{j=0}^{k-1}p_{-T+j}\right)\right]u(m_{-T+k})$$

For *m = R:*

(17) $$U^{R}=0$$

(18) $$u\left(m_{i}\right)=\{\begin{array}{c} \frac{-\lambda {\left(R-m_{i}\right)}^{\kappa }}{1-\kappa }\;m_{i}<R\;\\ \;\frac{{\left(m_{i}-R\right)}^{\omega }}{1-\omega }\;m_{i}\geq R\; \end{array}$$

(19) $$g\left(p_{i}\right)=\{\begin{array}{c} e^{-{\left(-\ln p_{i}\right)}^{\alpha }}\;m_{i}<R\;\\ \;e^{-{\left(-\ln p_{i}\right)}^{\delta }}\;m_{i}\geq R\; \end{array}$$

This is a rank-dependent model where small probability extreme outcomes are overweighted (*α* ∈ [0, 1], *δ* ∈ [0, 1]), and outcomes below the reference point have more influence than relative gains (here *λ* ∈ (1, 5)). Rather than using the status quo as the reference point, we used a non-zero reference point of £1.20. This enables the model to overweight small probability events at both extremes of the distribution, a parallel to skewness sensitivity.

Our models compare the utility of the lottery with the value of the sure amount (*S*) to generate a trial-by-trial probability of choosing the lottery over the sure amount, using a logistic/softmax function which allows for noise in action selection (by free parameter β).

(20) $$P_{choose\;gamble}=\frac{1}{1+e^{-\left(U-S\right)/\beta }}$$

We estimated best-fitting model parameters using maximum likelihood analysis, with optimisation implemented with a non-linear Nelder–Mead simplex search algorithm in Matlab (Matlab, Natwick, USA) and compared models using Group Bayes Factors, with the Akaike Information Criterion (AIC) (Akaike, 1974) and Bayesian Information Criterion (BIC) (Schwarz, 1978) providing approximations to the model evidence and penalising model complexity (Penny et al., 2004).

### fMRI—scanning parameters and preprocessing

We acquired gradient echo T2*-weighted echo-planar images (EPI) with blood-oxygen-level-dependent (BOLD) contrast, on a 3 Tesla head scanner (Magnetom Allegra, Siemens Medical). Imaging parameters were: 42 oblique transverse slices; slice thickness, 2 mm; gap between slices, 1 mm, repetition time TR = 3.1 s; echo time TE = 25 ms; field of view FOV = 192 × 192 mm^2^, matrix size 128 × 64 (RO × PE). We employed an EPI sequence optimised to avoid signal drop-out in the OFC using a combination of an increased spatial resolution in the read-out direction and a reduced echo time, as this generally enhances signal across a range of fronto-temporal regions (Weiskopf et al., 2007). During the same experimental session, a T1-weighted image was obtained for anatomical reference. To correct for geometric distortions induced in the EPIs at high field strength, we collected fieldmaps based on dual echo-time images (TE1 = 10 ms, TE2 = 12.46 ms), and processed these using the SPM8 fieldmap toolbox (Hutton et al., 2002) to produce a voxel displacement map indicating the field distortions. Images were realigned with the first volume, normalized to a standard EPI template, and smoothed using an 8 mm full-width at half-maximum Gaussian kernel. Unwarping was carried out using the routine in SPM8, correcting for distortions in each acquired image by combining the measured fieldmaps with estimated susceptibility-induced changes due to motion.

### fMRI—statistical analysis

Data were analyzed at the within-subject level with a single general linear model (GLM), with BOLD responses to each stimulus modelled as a box-car of duration 7.5 s (duration of stimulus presentation), time-locked to stimulus presentation and convolved with a hemodynamic response function. Intertrial interval (fixation cross) was 1–3 s. We constructed regressors to identify parametric responses to variance and skewness, identically modelled to the stimulus onset regressor, but with the height of the box-car modulated by the (mean-corrected) magnitude of lottery variance and skewness on each trial. Trials were split into positively and negatively skewed lotteries, and for each trial type we included both an unmodulated onset regressor, and also modulated regressors indicating variance, skewness, and the subject's choice (gamble or sure) per trial. Sure amount screens, keypresses, and resolution of gambles at the end of each block were also modelled to factor out BOLD activity unrelated to variables of interest. The experiment was conducted over one scanning period (the subject remained in the scanner throughout), but split into 3 scanner runs with brief breaks in between. We therefore account for session effects (e.g. scanner drift) in the design matrix by modelling each run separately. We also included subject-specific realignment parameters from the image preprocessing to account for motion-related artefacts in the images that were not eliminated in rigid-body motion correction. Beta values of linear contrasts for variance, (positive or negative) skewness, and choice, were estimated and entered into t-tests using random-effects analysis to provide group statistics.

At the between-subject level, we also included covariates derived from the behavioural model to indicate individual preferences for variance and skewness. This allows us to test two distinct hypotheses with regard to how preferences are integrated in the brain. We test either if inter-individual differences in behavioural preference modulate either the representation of the summary statistics themselves, or alternatively modulate choice-related activity. We used a cluster-defining voxel-wise threshold of p < 0.01, reporting whole-brain significant clusters family-wise error (FWE) corrected for multiple comparisons at p < 0.05, or significant voxels within a priori regions of interest (small-volume FWE-corrected at p < 0.05). Regions of interest based on previous studies comprise anterior insula/inferior frontal gyrus, ventral striatum and dorsomedial prefrontal cortex (Christopoulos et al., 2009; Mohr et al., 2010a; Preuschoff et al., 2006; Tobler et al., 2009). Percent signal change within a cluster is estimated with RFXplot (Gläscher, 2009). Figures show second-level SPM-T images thresholded at p < 0.005, superimposed upon a canonical image. Stereotactic coordinates are reported in MNI space (Mazziotta, 2001).

## Results

### Behaviour

Subjects (n = 23) distributed their choices between gamble and sure options throughout the course of the experiment (mean percentage of gamble choices = 53%, std. 14%). The sure option changed over the course of the experiment enabling us to decorrelate choice from the statistical features of interest. Additionally, as we focused on deconstructing risk (i.e. the distribution of outcomes), the expected value of the gamble was kept constant throughout (between £1.26 and £1.34). When the sure amount was greater, subjects therefore opted to gamble less often (mean percentage of gamble choices per sure amount level - 90p: 85%, std. 13%; 120p: 58%, std. 20%; 150p: 18%, std 14%). There were few error (missed) trials (4 +/− 0.5%), which are excluded from analyses. The mean correlation between choice and variance was − 0.009 (std 0.079) and the mean correlation between choice and skewness was 0.0035 (std 0.087).

#### Behavioural modelling

We independently manipulated variance and skewness, and predicted that individuals’ preferences would be sensitive to both summary statistics. As described in methods, we compared a mean-variance-skewness model (**MVS**) where individuals are allowed to express preferences for both variance and skewness, to a set of alternative decision models.

**Fig. 2:**
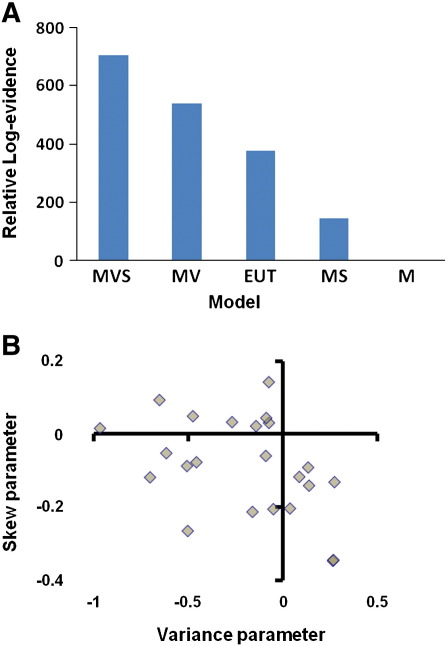
Behavioural modelling. A. Relative log-evidence for each of 5 models: mean only (M), mean-variance (**MV**), mean-skewness (**MS**), mean-variance-skewness (**MVS**) and power utility (**EUT**). Relative log-evidence (log-Group Bayes Factor) calculated as summed difference in log-evidence for each model relative to worst performing **M** model, across subjects. Model evidence is approximated by the Akaike information criterion (AIC), calculated as *AIC = 2.k − 2.ln(L)*, where *L* is the maximum likelihood estimate of the model and *k* is the number of free parameters. A higher score indicates a better model fit (higher model likelihood). There was strong evidence in favour of the **MVS** model in a fixed effects analysis of Group Bayes Factors (model posterior probability > 0.99 in favour of **MVS**). B. Parameter estimates from the **MVS** model reveal a range of preferences for variance (negative coefficent reflects variance aversion), and skewness (negative coefficient reflects aversion to positive versus negative skewness).

As predicted, a mean-variance-skewness (**MVS**) model provided a significantly better fit to the behavioural data than the 4 main alternative models (summed AIC: **M**: 4139; **MV**: 3599; **MS**: 3993; **MVS**: 3431; **EUT**: 3760; Group Bayes Factors (log-GBF relative to worst performing **M** model: **M**: 0; **MV**: 540; **MS**: 145; **MVS**: 708; **EUT**: 378 (Kass and Raftery, 1995; Raftery, 1995; Penny et al., 2004); **MVS** model posterior probability > 0.99 (very strong evidence in favour of **MVS**) (Fig. 2A). Similar results were obtained using the Bayesian Information Criterion (BIC) (Schwarz, 1978), an approach which penalises model complexity more severely than the AIC (log-GBF relative to **M** model calculated from BIC: **M**: 0; **MV**: 466; **MS**: 72; **MVS**: 561; **EUT**: 305). Here, we paid subjects for 18/180 trials during the entire experiment, motivated by a need to keep individuals engaged with the task. While paying for multiple trials has the potential to blunt risk-sensitivity, the fact that our risk-sensitive models were clearly superior to the risk-neutral (**M**) model demonstrates that risk-sensitivity was preserved, and suggests that participants assessed and treated each gamble individually.

Given our intuition that positive and negative skewness exert separate influences on behaviour, we also tested whether models with separate parameters for positively and negatively skewed gambles (i.e. one extra parameter than the **MVS** model) fit participants’ choices better than the three-parameter **MVS** model, and also whether models with preferences for variance and either positive or negative skewness fitted choice as well as the full **MVS** model. Again, the **MVS** model proved superior to these other models (Supplementary Fig. 2).

Although we mitigated severe probability distortion by constraining our gambles such that the smallest probability used was 0.1, it nevertheless is possible that behaviour attributed to skewness preference could be caused by probability weighting effects. To outrule this, we fit an additional model with probability weighting to our behavioural data, using the same specification as Hsu and colleagues (Hsu et al., 2009), with power utility and a 1-parameter (Prelec) probability weighting function. Although this outperformed a power utility model without probability weighting, it was vastly inferior to the mean-variance-skewness model (probability weighting model AIC: 3674, log Group Bayes factor **MVS** vs probability weighting = 243). We also fit a cumulative prospect theory (**CPT**) model, with the reference point set at the £1.20 sure amount rather than the status quo of £0. This allows for potential overweighting of small probability outcomes at both extremes of the distribution, similar to skewness preference. The **MVS** model outperformed the **CPT** model (log Group Bayes factor **MVS** vs **CPT**: 236; Supplementary Fig. 2).

We next used our winning **MVS** model to provide subject-specific preferences for variance and skewness. Parameter estimates from the **MVS** model showed that 16/23 subjects were averse to variance (average variance preference: −0.20; s.e.m. 0.07), and 15/23 were averse to positive skewness (average skewness preference: −0.09; s.e.m. 0.03) (Fig. 2B). Beta (temperature) values for the logistic function were low, indicating that choices were well partitioned by the linear model (average beta = 0.14; s.e.m. 0.01). Some subjects had strong skewness preference but were insensitive to variance, other subjects were indifferent to skewness. 8/23 showed a negative variance and skewness parameter, which corresponds to a (locally) sigmoid utility function. There was a weak negative correlation between variance and skew-preference (*r*^2^ = 0.17; p = 0.05). No individuals in our sample were both variance and positive-skew-seeking. The **MVS** model was at least as good as the **MV** and **MS** alternatives in the majority of individual cases, outperforming the **MS** model in 19/23 subjects and the **MV** model in 17/23 subjects.

It is possible that subjects might switch their behavioural preferences from preferring positive to preferring negative skewness over the course of the experiment. We checked this possibility by separately fitting the **MVS** model per subject to the first and second half of the data. We found no evidence that individuals reverse their preference for skewness during the experiment, with 15 subjects starting and finishing with preference for negative skewness, 5 subjects starting and finishing with preference for positive skewness, and only 3 subjects reversing preference from negative to positive skew seeking (20/23 subjects with no switch in preference, binomial test = n.s.). We explored this question further, and tested if there was any systematic shift in preference at all. There was no significant change in the estimated variance preference parameters from the 1st to the 2nd half of the session across subjects (paired *t*-test, p = .75). However there was a change in skew-preference (paired *t*-test, p = 0.005). Consequently, for the imaging analysis, we use individuals’ average behavioural preferences estimated across the experimental session. Note that any change in preference over time will count against our analysis by introducing noise into the data, rendering it less likely to detect a significant result, and also mean that we may underestimate the true effect size of some reported correlations.

### Functional imaging

#### Responses to variance and skewness

**Fig. 3:**
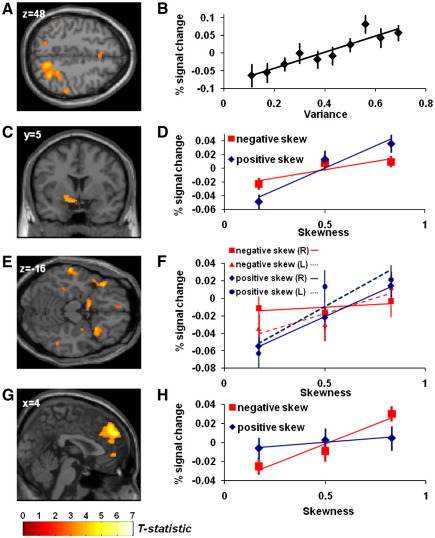
Responses to summary statistics. Figure shows second-level SPM-T images thresholded at p < 0.005, superimposed upon a canonical image. A. Linear correlation between PPC activity and variance (peak coord: 32, −60, 50; p = 0.003, whole-brain corrected). B. Estimated percent signal change, averaged activity over all voxels within PPC cluster. C. Correlation between increasing positive skewness and BOLD signal in ventral striatum (peak coord: −10, 4, −14; p = 0.033, small volume corrected). D. Estimated percent signal change, averaged activity over all voxels within ventral striatum cluster, for positive and negative skewed gambles. E. There was also positive correlation seen in bilateral anterior insula (peak coords: right—30, 16, -14, p = 0.021; left—−40,24, −16, p = 0.017; small volume corrected). F. Estimated percent signal change for positive and negatively skewed gambles, averaged activity over all voxels within right and left anterior insula clusters (plotted separately). G. Correlation between increasing negative skewness and BOLD signal in DMPFC (peak coord: 4, 44, 36; p < 0.001, whole-brain corrected). H. Estimated percent signal change, averaged activity over all voxels within ventral striatum cluster. Error bars on correlation plots show standard error. All statistical inference performed in SPM (reported in the main text and supplementary tables), thus separate correlation strength and effect size not re-calculated from plotted correlations, with relationships shown for illustrative purposes.

Having established that a **MVS** model best explained participants’ choice behaviour, we next asked whether statistical components of this model have a distinct neural representation. In line with our predictions, brain activity in right PPC (peak MNI coord: 32, −60, 50; p = 0.003, cluster extent = 1318 voxels) showed a significant correlation with lottery variance on each trial, irrespective of choice (Figs. 3A and B, Supplementary Table 2). No brain activity negatively correlating with variance survived family-wise error-correction.

**Fig. 4:**
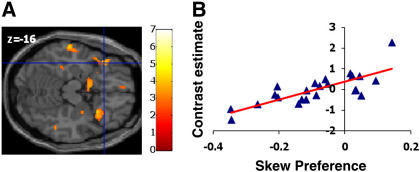
Correlation of skew-evoked activity with individual preferences. A. Within the anterior insula regions showing a correlation in signal with positive skewness, the left anterior insula shows a significant positive correlation with individual skew-preference (peak coord: −36, 24, −16; p = 0.007, small-volume corrected). B. Plot of behavioural model (**MVS**) skewness parameter estimate against neural contrast estimate for positive-skew related activity.

In contrast to a segregated representation of variance, we observed a distributed encoding of skewness. Using our pair of regressors representing stimulus-evoked BOLD activity modulated by the degree (magnitude) of lottery skewness, for positive and negatively skewed trials respectively, we tested both whether there were regions encoding the full range of skewness on a linear scale, and whether there were regions whose activation depended solely on the degree of positive or negative skewness. No single area linearly correlated with the full range of skewness (i.e. both increasing activation for greater positive skewness, and decreasing activation for greater negative skewness), which we assessed by testing for a conjunction between activity for positive and negative skewness (at p < 0.01 voxelwise threshold). Instead, we found dissociable cortical and subcortical regions individually correlating with positive and negative skewness. As positive skewness increased (a small chance of a better than average outcome) so too did BOLD signal in ventral striatum (peak voxel MNI coord: −10, 4, −14; p = 0.033 (small volume corrected), cluster extent = 228 voxels), and bilateral anterior insula extending into inferior frontal gyrus (IFG) (on right: peak voxel MNI coord: 30, 16, −14; p = 0.021 (small volume corrected), cluster extent = 234 voxels. on left: peak voxel MNI coord: −40, 24, −16; p = 0.017 (small volume corrected), cluster extent = 67 voxels.) (Figs. 3C and 4, Supplementary Table 2), a priori regions of interest where risk-related activity has been seen in previous studies (Schultz et al., 2008). In contrast, negative skewness correlated with activity in medial prefrontal cortex (peak voxel MNI coord: 4, 44, 36; p < 0.001, cluster extent = 1673 voxels) (Fig. 3E, Supplementary Table 2). There were no areas surviving correction that correlated with decreasing positive or decreasing negative skewness (i.e. greater BOLD signal for less skewed lotteries). Ventral striatum and prefrontal cortex expressed dissociated responses to positive and negative skewness (Figs. 3D and F). No areas correlated with increasing magnitude of skewness irrespective of sign (i.e. more active for skewed than symmetrical lotteries, irrespective of whether the outliers were better or worse than the average) survived correction.

Given that the **MVS** model specifies value as a linear mixture of variance and skewness, we would expect that any regions expressing activity correlating with gamble utility would be expected to also show a partial correlation with both variance and skewness. Conversely, the fact that we do not see common regions correlating with variance and skewness would argue against a unitary representation of lottery value. However, this may be due to reduced detection power in an additive mixture of random variables, thus we also tested directly for a representation of value. We ran separate GLMs to identify regions correlating with the trial-by-trial utility of the lottery (stimulus value; **MVS** model), the difference in utility of lottery vs. sure amount (relative stimulus value), and utility of the chosen–utility of the unchosen option (relative action value). No areas correlated with stimulus value, even within regions of interest (variance and skew-sensitive areas, or prefrontal cortex) at an uncorrected (p < 0.001) threshold. For relative stimulus value, we observed a 5-voxel cluster at uncorrected significance (p < 0.001, voxel level) in right ventromedial OFC. Relative action values were reflected in robust activation in a network of regions comprising bilateral precentral sulcus extending into supplementary motor area, and bilateral inferior parietal lobe (p < 0.001, corrected). As might be expected, these regions form a typical motor/motor preparatory network (Cunnington et al., 2002).

#### Integration with variance and skewness preferences

We considered whether regions encoding the statistics of lottery outcomes also integrated this information with individuals’ tastes for risk. These variance or skewness-encoding regions could express different sensitivities to these statistics depending upon an individual's risk preferences. Thus we tested whether areas expressing variance and skewness-related activity altered in sensitivity (correlation effect size) to these statistics in a manner that correlated with subjects’ preferences, as estimated from the **MVS** model. In regions encoding either positive or negative skewness, we identified a significant interaction between BOLD activity for positive skewness and subject-specific skew-preference (peak voxel MNI coord: -36, 24, −16; p = 0.007, cluster extent = 80 voxels) in anterior insula. Thus, the more positive a subjects’ skew parameter (preference for positive over negative skewness) the stronger the correlation with skewness in this area (Fig. 4, Supplementary Table 3). There was no significant correlation surviving correction between behavioural preference and skewness-evoked activity in other regions. By contrast, the posterior parietal area correlating with variance did not express differential activity that co-varied with subject-specific variance preference (masked for voxels expressing variance-related activity, no significant voxels at mask threshold 0.05 uncorrected).

#### Choice-related activity

**Fig. 5:**
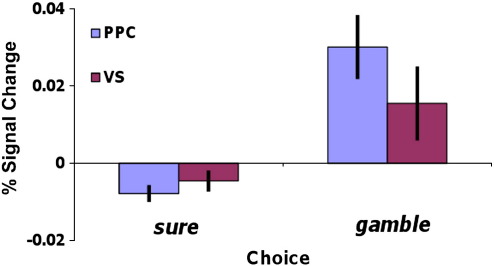
Choice-related activity. Both ventral striatum and posterior parietal cortex (PPC) show significantly greater BOLD signal for gamble versus sure choices (ventral striatum peak coord: −8, 4, −10; p = 0.016, small-volume corrected; PPC peak coord: 26, −60, 54; p = 0.008, small-volume corrected).

Information about summary statistics informed individuals’ choices, hence we next asked whether activity in regions responsive to variance and skewness also correlated with choice. There was a significant effect of choice (the choice regressor expressing the effect of gamble > sure) within variance-sensitive PPC (peak voxel MNI coord: 26, −60, 54; p = 0.008, cluster extent = 547 voxels, small volume-corrected for variance-related areas of activity) (Fig. 5), in skew-sensitive ventral striatum (peak voxel MNI coord: −8, 4, −10; p = 0.016, small volume-corrected for skewness-related areas of activity) and medial prefrontal region (peak voxel MNI coord: 6,44,18; p = 0.049, small-volume corrected for skewness-related areas of activity) (Supplementary Table 4). In addition to these areas, across the whole-brain, a network of regions showed greater BOLD signal for gamble than sure choices (Supplementary Table 5), including ventral striatum, prefrontal, occipital and bilateral posterior parietal cortex. No areas showed the opposite pattern (sure > gamble signal).

**Fig. 6:**
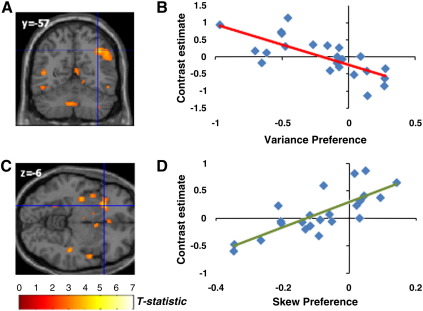
Coupling of choice and neural activity in PPC and anterior insula depends upon subject-specific risk preferences. A. Correlation between PPC activity for choice (gamble > sure) and individual variance-aversion (peak coord: 36, −58, 42; p = 0.035, whole-brain corrected). B. Contrast estimate (for gamble > sure choice), from peak coordinate (indicated by cross-hairs), correlated with behavioural variance preference parameter, with greater activity for gamble vs sure choices in variance-averse individuals, but greater activity for sure vs gamble choices in variance-seeking individuals. C. Correlation between anterior insula activity for choice (gamble > sure) and individual skew-preference (peak coord: left: −24, 22, −6, p = 0.033; right: 54, −4, −12, p = 0.039; small-volume corrected). D. Contrast estimate (for gamble > sure choice), from peak coordinate (indicated by cross-hairs), correlated with behavioural skewness-preference parameter, with greater activity for gamble vs sure choices in positive skew-seeking individuals. Within this cluster, there was also a significant correlation between variance-seeking and choice-related activity (at coord: 46, −4, −14; p = 0.040, small volume corrected for skew-sensitive voxels).

If activation correlating with choice within risk-sensitive regions influences action selection, a strong expectation is that this choice-coupled neural activity should also be modulated by individual risk preferences. This provides an alternative mechanism for risk preferences to influence decisions, rather than only by altering neural sensitivity to the stimulus dimensions of variance and skewness. For example, in regions sensitive to variance one might predict an enhanced correlation of BOLD signal with choice (gamble > sure) in more variance-averse individuals. Within the variance-sensitive PPC region, we observed just such an effect (peak voxel MNI coord: 36, −58, 42; p = 0.035, cluster extent = 656 voxels). Thus, for subjects with strong variance aversion there was greater activity for a gamble choice than a sure choice (Figs. 6A and B). Clusters in right supplementary motor area (SMA), posterior cingulate, and occipital lobe also showed a similar relationship (Supplementary Table 6). Performing the same analysis for skewness revealed that left anterior insula and right mid-insula express a positive correlation between choice and skew-preference (peak voxel MNI coord: −24, 22, −6; p = 0.033, small-volume corrected, cluster extent = 666 voxels) (Figs. 6C and D, Supplementary Table 7). Moreover, the same right insula region showing an interaction between choice-related activation and skew-preference also showed a positive correlation between choice-related activation and variance-preference (at voxel MNI coord: 46, −4, −14; p = 0.040 small volume corrected for skew-sensitive voxels, Supplementary Table 6), suggesting that right anterior insula activity integrates both variance- and skew-preferences to influence choice.

## Discussion

We show that individuals’ choices are sensitive to both the spread (variance), and the shape and asymmetry of a distribution of possible outcomes (skewness). Moreover, these different risk dimensions have distinct neural representations, providing strong evidence that the brain adopts a ‘summary statistic’ approach to outcome evaluation (Rangel et al., 2008).

Our paradigm substantially differs from previous neuroeconomic approaches to risk, as we independently manipulate statistical features of a distribution of outcomes. Risk typically is approximated by variance (Christopoulos et al., 2009; McCoy and Platt, 2005; Preuschoff et al., 2006; Tobler et al., 2007). This neglects other psychologically salient features, such as small chances of much better (positive skewness) or worse (negative skewness) than average outcomes (Coombs, 1960; Harvey and Siddique, 2000; Jullien and Salanie, 2000). Contrasting with alternative risk-measures focusing only on the chance of poor outcomes (Bawa, 1975; Fishburn, 1984), we find participants influenced by both negative and positive skewness in addition to variance.

Skewness-preference permits simultaneous ‘risk-aversion’ and ‘risk-seeking’, inexplicable by variance-sensitivity alone (Garrett and Sobel, 1999). On average, our participants were both variance and positive skew-averse, disliking uncertainty (outcome dispersion) and preferring gambles with mostly above average rewards but risking a small chance of low payoffs. An example of negative skew preference would be to prefer property investment, with reasonable average yields but a small chance of heavy losses, to the opportunity of investing in oil exploration, with potentially high yields but a large probability of not recouping one's original investment. In contrast with normative theoretical predictions of positive skew preference in variance averse individuals (Scott and Horvath, 1980), we find relative negative skew preference and variance aversion (on average) in our sample of participants. However, in experiments where preferences for lotteries with different variance and skewness have been systematically examined, individuals in fact exhibit heterogeneous behaviour.

Several studies have reported predominantly positive skew-seeking behaviour (Alderfer, 1970; Coombs and Pruitt, 1960), or used positive skew preference to explain gambling behaviour (Garrett and Sobel, 1999; Golec and Tamarkin, 1998). Other studies have reported varied preferences, with different participants showing positive or negative skew-seeking as is the case in our study (Lopes, 1984), or negative skew preference on average (Lichtenstein, 1965). Skewness has also been shown to influence perceived riskiness in different directions (Coombs and Bowen, 1971), and negative skew-seeking investment behaviour is also common in investors (Taleb, 2004; Tan, 1991). Here we systematically examine these separate influences by experimental design, both showing that the overall shape of the outcome distribution drives choice and independently evokes neural activity. Together this supports the idea that ‘risk preference’ is not a unitary measure, either of behaviour or in terms of activity it is likely to evoke in the brain. While there are no clear biological restrictions on the range of preferences individuals are allowed to exhibit in a summary statistic framework, it is nonetheless interesting to speculate on the reasons why these studies report different preferences for variance and skewness. Distorted estimates of very small probabilities are likely to have an additional impact on choice, and some previous studies have used extremely unlikely events when demonstrating skew-preferences. Many previous studies also present mixed gambles (with both possible financial losses and gains), rather than presenting decisions purely in the gain domain as in the current study. This raises the possibility that contextual frame (loss or gain) of the decision could additionally influence predilections for risk.

Consistent with our behavioural finding of sensitivity to different elements of risk, we also find distributed risk representation in the brain. Variance was linearly encoded in PPC, concurring with single unit and fMRI data showing enhanced PPC activity during risky decision making (Huettel et al., 2005; Mohr et al., 2010a; Platt and Glimcher, 1999). Parietal cortex represents numerical range (Piazza et al., 2007) and expresses an interaction between number and space (Hubbard et al., 2005), suggesting that variance representation in PPC reflects an intuitively spatial evocation of the spread of an outcome distribution. This may explain the absence of PPC activity when risk is varied by altering win probability, rather than manipulating the range of outcome amounts (Preuschoff et al., 2006). While possible that PPC expresses an effect consequent upon increasing risk, such as enhanced attention (Behrmann et al., 2004), we find a specific effect for variance rather than skewness, despite both influencing risk perception.

Skewness-related activity in DMPFC, insula, and striatum encodes a dimension independent of variance, emphasising that risk is not synonymous with variance alone. Anterior insula and DMPFC are consistently implicated in risk-processing (Bach et al., 2009; Behrens et al., 2007; Christopoulos et al., 2009; Critchley et al., 2001; Engelmann and Tamir, 2009; Kuhnen and Knutson, 2005; Mohr et al., 2010b; Smith et al., 2009; St Onge and Floresco, 2009; Tobler et al., 2007), and ventral striatum manifests immediate and delayed responses to probability (Preuschoff et al., 2006) and reward magnitude (Knutson et al., 2005; Yacubian et al., 2007). These prior observations may have tapped into a combination of risk elements, while here we dissociate variance and skewness. Previously observed non-linear ventral striatum activation for probability (Hsu et al., 2009) could be explained by positive skewness encoding as these variables are correlated when binary outcomes are used. It is also possible that the converse is true, although less likely given that we employ multiple outcomes. Moreover, there is no clear model of how multiple outcomes would be encoded, indeed one might expect neural representation of both ‘win’ and ‘loss’ outcomes assuming separate encoding of amounts and probabilities. While agnostic as to the exact coding of the summary statistics of outcome distributions (e.g. variance, standard deviation and coefficient of variation are correlated measures of spread), critically we find responses to dispersion versus asymmetry of outcomes evokes activity in anatomically separate networks.

We identify responses to positive skewness in ventral striatum, anterior insula, and IFG, but to negative skewness in DMPFC. This distinction parallels related findings that DMPFC encodes the probability of loss (Smith et al., 2009; Xue et al., 2010) and could explain why studies do not report risk-correlation in ventral striatum when skewness is controlled (Christopoulos et al., 2009; Tobler et al., 2009). Consistent with our data, PPC insula and DMPFC activation was also reported in a study with choices between multiple-outcome lotteries (Venkatraman et al., 2009), potentially reflecting preferences for different kinds of outcome distribution. Our finding of separable anatomic regions correlating with positive and negative skewness does not predict that subjects would have distinct attitudes toward positive and negative skewness. While this is a plausible prediction we find no evidence for a separate expression of preference in these regions. The skewness parameter from the **MVS** model reflects how much subjects value positive relative to negative skewness in a gamble. Given that we can approximate subjects’ behaviour with a single skew parameter, it is equally likely that skew-preference arises from an interaction between brain areas. In order for the **MVpSnS** model to win (in terms of predicting choice), our participants would need to have very different sensitivities for positive and negative skewness, or express skewness intransitivities such as liking symmetric above any skewed gambles. We acknowledge that it is possible that within a larger behavioural sample we would have sufficient data to demonstrate such different sensitivities for positive and negative skewness.

A recent paper (Wu et al., 2011) has also examined skewness, investigating neural responses to the presentation of 4 repeatedly presented mixed gambles (high variance, low variance, positively skewed, negatively skewed) in a passive viewing (no choice) paradigm. In line with our present findings, they report greater ventral striatal activity for positive versus negatively skewed gambles and anterior insula sensitivity to skewness. There are several important differences and limitations in the aformentioned study compared to the present experiment. Critically, our subjects made active choices allowing a distinction between processes supporting risk quantification and evaluation from choice. We did not present outcomes of gambles on each trial, thus could outrule feedback-related activity. In addition, we employ an orthogonal parametric design presenting a range of gambles with different variance and skewness, avoiding a potential confound between skewness and high variance and the limitation of using solely 4 stimuli in a cognitive paradigm. These design features enables us to accurately and independently measure behavioural and neural sensitivity to each risk dimension. Interestingly, Wu at al elicited ratings of arousal, perceived risk and preference for each of the 4 lotteries and found that these ratings are not commensurate, highlighting a disparity between the gambles that subjects preferred and gambles that evoked an affective response. Despite preferences in their sample being very different from our present participants, there is a similarity in neural responses, with anterior insula being active for positively skewed gambles in our negative-skew preferring subjects, whereas it was most active for skewed versus symmetric gambles in Wu's study where subjects exhibit converse preferences.

Medial PFC and striatum are reciprocally connected (Croxson et al., 2005; Sesack et al., 1989), project to prefrontal and pre-motor areas involved in action planning and execution (Haber, 2003), and insular cortex (Guldin and Markowitsch, 1983; Shi and Cassell, 1998). These connections can mediate information transmission between areas translating different features of the outcome distribution into summary statistics, and areas integrating this information with context and individual risk preferences. This distributed network for risk evaluation echoes distributed neural processing in vision, where discrete visual dimensions (colour, motion, form), are processed in segregated networks (Courtney and Ungerleider, 1997).

Anterior insula and IFG activity is modulated by individual taste for risk, expressing greater activity for positively skewed gambles when individuals prefer positive skewness, and showing a correlation with choice also dependent upon individual skewness and variance-preference. This supports the idea of these areas evaluating risk and subsequently promoting or inhibiting risk-taking (Christopoulos et al., 2009; Engelmann and Tamir, 2009; Paulus et al., 2003; Xue et al., 2010). The insula is suited to perform such integration, central in representing interoceptive states consequent upon perception of risk (Craig, 2009; Singer et al., 2009), with motoric basal ganglia and pre-motor projections, and to value-comparison regions in orbitofrontal cortex (Augustine, 1996; FitzGerald et al., 2009; Plassmann et al., 2007). We observed greater loading for skewness than variance in anterior insula, generated by a range of skewness-preference in our subjects, as opposed to previous studies where skewness was fixed (Christopoulos et al., 2009; Tobler et al., 2009). We also decorrelated choice from risk, whereas previous studies may have detected risk anticipation contingent on choice rather than the process of quantifying decision statistics (Christopoulos et al., 2009; Kuhnen and Knutson, 2005; Preuschoff et al., 2006; Tobler et al., 2009; Xue et al., 2010). Anticipation of chosen risk could recruit insula activity, explaining consistent reports of (risk-attitude dependent) activity in this region.

PPC and ventral striatum activity also correlated with choice, with the strength of correlation in PPC dependent upon the degree of variance-aversion, corroborating previous findings (Weber and Huettel, 2008). Striatum responded to both positive skewness and gamble choice, although subjects mostly avoided positively skewed gambles. One possibility here is that striatum could encode statistical properties of a gamble and independently engender action following integration of risk preferences and statistical information. Note that variance preferences are also significant in driving choice, hence could also influence striatal activity. Alternatively, striatum could exert a negative influence on the choice to gamble. The pattern of activity we observed in PPC would be expected in a region inhibiting risky choice, as the strongest coupling occurs in variance-averse individuals. This region overlaps the medial intraparietal area, integral to motor intention (Andersen and Cui, 2009; Grefkes et al., 2004), thus parietal cortex might promote safe choices via polysynaptic links to basal ganglia and premotor regions (Clower et al., 2005; Tanne-Gariepy et al., 2002). The PPC has direct insula connections (Cavada and Goldman-Rakic, 1989), thus may also directly pass quantitative information about variance to anterior insula.

It is interesting that a network of brain regions demonstrates greater activation for a gamble than a sure choice, as has been reported in previous studies (Christopoulos et al., 2009; Matthews et al., 2004; Weber and Huettel, 2008). This indicates that recruitment of neural regions is different when generating gamble versus sure choices. This is contrasts with the simplifying assumption portrayed in our behavioural models, where the process of choice is modelled as a softmax comparison between equally-weighted utilities for the lottery and sure amount. While we make no assumptions here about the neural processes underlying choice generation, similar differential activation in striatum has been related to the acceptance or rejection of a default action (Yu et al., 2010) and in prefrontal cortex during the selection of ‘exploratory’ actions (Daw et al., 2006).

In this study, we consider two plausible ways in which individual preferences could modulate neural activity. We first find preferences modulating stimulus-evoked activity, with individuals with stronger preference for skewness demonstrating increased insula sensitivity to skewness. Secondly, we show variance and skewness preferences modulate action-related neural activation, as individuals with stronger preferences have greater coupling between neural activity and choice within risk-sensitive regions. Overall, this provides evidence that preferences modulate both stimulus-evoked and choice-related activity.

## Conclusion

Classical utility theory assumes values are assigned to possible states of the world, then weighted by probability (Machina, 2005). We provide evidence for an alternative hypothesis, that the brain is adapted to decompose decisions into summary statistics reflecting dispersion and asymmetry of outcome distributions. Compartmentalizing independent stimulus properties to deconstruct neural networks supporting perception is well established in sensory neuroscience. Similarly, we find that contrary to common assumption, risk is not monolithic but can be decomposed into separate dimensions which have segregated representations in the brain. Strikingly, neural activity in risky choice also reflects individuals’ tastes for different features of an outcome distribution, an evaluation and integration supported by dissociable neural regions involved in risk processing.
