# Supplementary material for: Deconstructing risk: Separable encoding of variance and skewness in the brain
Source: Neuroimage. 2011 Oct 15;58(4):1139–49. doi: 10.1016/j.neuroimage.2011.06.087 (PMC3176914; doi:10.1016/j.neuroimage.2011.06.087)
Supplement: Supplementary file 1 — Supplementary Materials [file mmc1.doc]

**Supplemental Material**


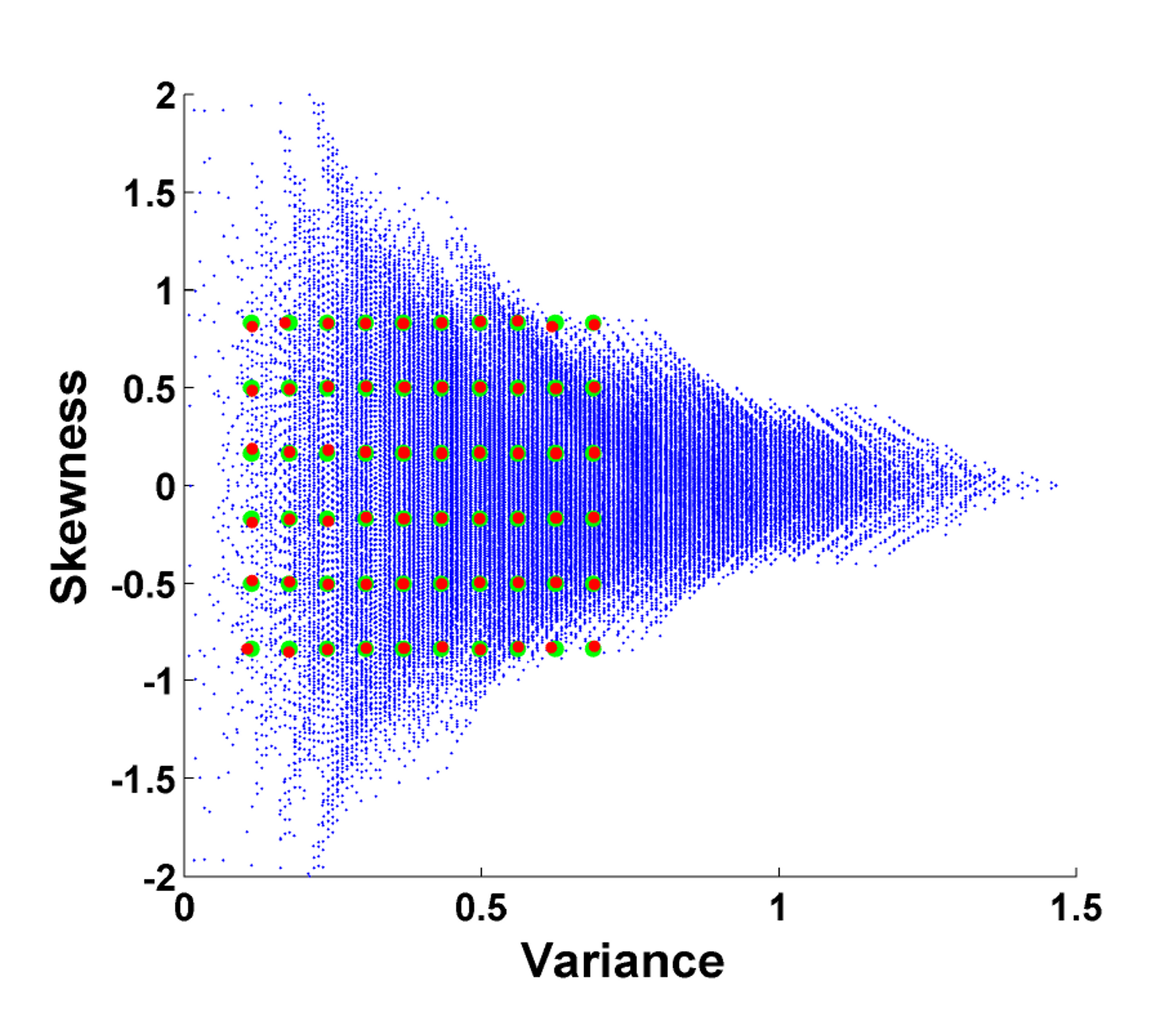


**Supplementary Figure 1: Construction of stimulus set**

**
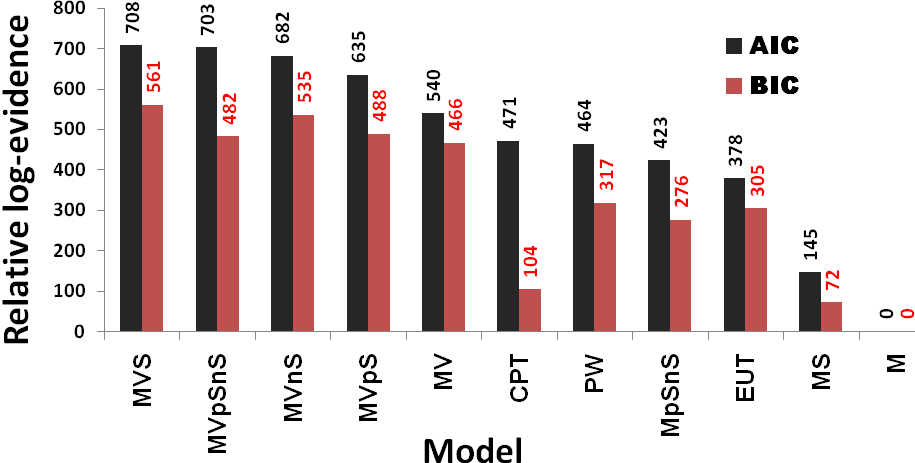
**

**Supplementary Figure 2: Comparison of additional behavioural models**

**Supplementary Figure Legends**

**Supplementary Figure 1:**

Figure of stimulus set construction, plotted as variance against skewness of lottery outcomes. Blue dots represent all possible gambles, constructed with EV = £1.26-£1.34, according to stimulus set constraints. Green dots represent desired array of stimuli, and red dots are the actual selected stimuli (n=60) for use in experiment, with independent manipulation of variance and skewness.

**Supplementary Figure 2:**

Comparison of models including: mean only (**M**), mean-variance (**MV**), mean-skewness (**MS**), mean-variance-skewness (**MVS**), power utility (**EUT**), mean-variance-positive skewness (**MVpS**), mean-variance-negative skewness (**MVnS**), mean-positve skewness-negative skewness (**MpSnS**), mean-variance-positive skewness-negative skewness (**MVpSnS**), probability weighting (**PW**) and cumulative prospect theory (**CPT**). Relative log-evidence, with model evidence approximated by either AIC or BIC to penalise for model complexity, calculated against worst performing **M** model, across subjects, and are given above each model. A higher relative log-evidence indicates a better model fit (higher model likelihood).

**SUPPLEMENTARY TABLE 1**

**Table of lotteries used in experiment:** 60 lotteries were generated to form the stimulus set for this experiment, orthogonal in variance and skewness by design. Lottery outcomes were between 20p and 260p, with associated probabilities varying in 0.1 intervals. The amounts and probabilities used for each lottery are shown (0 indicates that amount not shown in lottery). The expected value (pence), variance ((pence)^2^ x 10^-4^) and skewness of each lottery are shown in the last 3 columns.

|  | **Amounts (p)** | | | | | | | | | | | | | |  |  |  |
| --- | --- | --- | --- | --- | --- | --- | --- | --- | --- | --- | --- | --- | --- | --- | --- | --- | --- |
| **Lottery number** | **0** | **20** | **40** | **60** | **80** | **100** | **120** | **140** | **160** | **180** | **200** | **220** | **240** | **260** | **Expected value** | **Variance** | **Skewness** |
| **1** | 0 | 0 | 0 | 0.1 | 0 | 0.2 | 0.1 | 0.2 | 0.4 | 0 | 0 | 0 | 0 | 0 | 130 | 0.11 | -0.83 |
| **2** | 0 | 0 | 0 | 0.1 | 0 | 0.1 | 0.2 | 0.4 | 0 | 0.2 | 0 | 0 | 0 | 0 | 132 | 0.11 | -0.49 |
| **3** | 0 | 0 | 0 | 0 | 0.2 | 0.1 | 0.2 | 0.1 | 0.3 | 0.1 | 0 | 0 | 0 | 0 | 130 | 0.11 | -0.19 |
| **4** | 0 | 0 | 0 | 0 | 0.1 | 0.3 | 0.1 | 0.2 | 0.1 | 0.2 | 0 | 0 | 0 | 0 | 130 | 0.11 | 0.19 |
| **5** | 0 | 0 | 0 | 0 | 0.2 | 0 | 0.4 | 0.2 | 0.1 | 0 | 0.1 | 0 | 0 | 0 | 128 | 0.11 | 0.49 |
| **6** | 0 | 0 | 0 | 0 | 0.1 | 0.1 | 0.5 | 0.1 | 0 | 0.1 | 0.1 | 0 | 0 | 0 | 130 | 0.11 | 0.81 |
| **7** | 0 | 0 | 0.1 | 0 | 0.2 | 0 | 0.1 | 0.1 | 0.5 | 0 | 0 | 0 | 0 | 0 | 126 | 0.18 | -0.85 |
| **8** | 0 | 0 | 0 | 0.1 | 0.2 | 0 | 0.1 | 0.1 | 0.3 | 0.2 | 0 | 0 | 0 | 0 | 132 | 0.18 | -0.49 |
| **9** | 0 | 0 | 0.1 | 0 | 0 | 0.1 | 0.1 | 0.6 | 0 | 0 | 0 | 0.1 | 0 | 0 | 132 | 0.18 | -0.17 |
| **10** | 0 | 0 | 0.1 | 0 | 0 | 0 | 0.6 | 0.1 | 0.1 | 0 | 0 | 0.1 | 0 | 0 | 128 | 0.18 | 0.17 |
| **11** | 0 | 0 | 0 | 0.1 | 0.1 | 0.1 | 0.2 | 0.3 | 0.1 | 0 | 0 | 0.1 | 0 | 0 | 128 | 0.18 | 0.49 |
| **12** | 0 | 0 | 0 | 0.1 | 0 | 0.1 | 0.6 | 0 | 0 | 0.1 | 0 | 0.1 | 0 | 0 | 128 | 0.17 | 0.83 |
| **13** | 0 | 0.1 | 0 | 0 | 0.2 | 0 | 0.1 | 0.2 | 0.2 | 0.2 | 0 | 0 | 0 | 0 | 126 | 0.24 | -0.84 |
| **14** | 0 | 0 | 0.1 | 0.1 | 0 | 0 | 0.3 | 0.1 | 0.1 | 0.2 | 0.1 | 0 | 0 | 0 | 132 | 0.24 | -0.50 |
| **15** | 0 | 0 | 0.1 | 0 | 0.1 | 0.2 | 0 | 0.1 | 0.4 | 0 | 0 | 0.1 | 0 | 0 | 132 | 0.24 | -0.18 |
| **16** | 0 | 0 | 0 | 0.2 | 0 | 0.2 | 0.2 | 0.1 | 0 | 0.1 | 0.2 | 0 | 0 | 0 | 128 | 0.24 | 0.18 |
| **17** | 0 | 0 | 0.1 | 0 | 0.1 | 0.1 | 0.1 | 0.5 | 0 | 0 | 0 | 0 | 0.1 | 0 | 128 | 0.24 | 0.50 |
| **18** | 0 | 0 | 0 | 0 | 0.2 | 0.4 | 0.1 | 0 | 0 | 0.1 | 0.1 | 0.1 | 0 | 0 | 128 | 0.24 | 0.83 |
| **19** | 0 | 0.1 | 0 | 0.1 | 0.1 | 0 | 0 | 0.1 | 0.4 | 0.1 | 0.1 | 0 | 0 | 0 | 132 | 0.31 | -0.83 |
| **20** | 0 | 0 | 0.2 | 0 | 0.1 | 0.1 | 0 | 0 | 0.4 | 0.1 | 0.1 | 0 | 0 | 0 | 128 | 0.31 | -0.50 |
| **21** | 0 | 0.1 | 0 | 0 | 0.1 | 0.1 | 0.4 | 0 | 0 | 0 | 0.3 | 0 | 0 | 0 | 128 | 0.31 | -0.16 |
| **22** | 0 | 0 | 0.1 | 0 | 0.3 | 0 | 0.2 | 0.1 | 0 | 0 | 0.3 | 0 | 0 | 0 | 126 | 0.30 | 0.17 |
| **23** | 0 | 0 | 0 | 0.1 | 0.1 | 0.4 | 0 | 0 | 0.1 | 0.1 | 0 | 0.2 | 0 | 0 | 132 | 0.31 | 0.50 |
| **24** | 0 | 0 | 0 | 0 | 0.4 | 0.2 | 0.1 | 0 | 0 | 0.1 | 0 | 0.2 | 0 | 0 | 126 | 0.30 | 0.83 |
| **25** | 0.1 | 0 | 0.1 | 0 | 0 | 0 | 0.2 | 0.1 | 0.4 | 0 | 0 | 0.1 | 0 | 0 | 128 | 0.37 | -0.83 |
| **26** | 0 | 0 | 0.3 | 0 | 0 | 0 | 0.2 | 0 | 0 | 0.5 | 0 | 0 | 0 | 0 | 126 | 0.37 | -0.50 |
| **27** | 0 | 0 | 0.2 | 0.1 | 0 | 0 | 0.1 | 0.3 | 0 | 0.1 | 0.1 | 0.1 | 0 | 0 | 128 | 0.37 | -0.17 |
| **28** | 0 | 0 | 0.1 | 0 | 0.3 | 0 | 0.2 | 0 | 0 | 0.2 | 0 | 0.2 | 0 | 0 | 132 | 0.37 | 0.17 |
| **29** | 0 | 0.1 | 0 | 0 | 0 | 0.3 | 0.2 | 0.2 | 0 | 0 | 0.1 | 0 | 0 | 0.1 | 130 | 0.37 | 0.50 |
| **30** | 0 | 0 | 0.1 | 0 | 0.2 | 0.2 | 0.2 | 0 | 0 | 0.2 | 0 | 0 | 0 | 0.1 | 126 | 0.37 | 0.83 |
| **31** | 0.1 | 0 | 0.1 | 0.1 | 0 | 0 | 0.1 | 0 | 0.1 | 0.5 | 0 | 0 | 0 | 0 | 128 | 0.43 | -0.83 |
| **32** | 0.1 | 0 | 0.1 | 0 | 0.1 | 0 | 0.2 | 0.1 | 0 | 0.3 | 0 | 0.1 | 0 | 0 | 126 | 0.43 | -0.50 |
| **33** | 0 | 0 | 0.2 | 0.2 | 0 | 0 | 0 | 0.2 | 0 | 0.1 | 0.3 | 0 | 0 | 0 | 126 | 0.43 | -0.16 |
| **34** | 0 | 0.1 | 0 | 0.1 | 0 | 0.1 | 0.2 | 0.2 | 0.1 | 0 | 0 | 0 | 0.2 | 0 | 134 | 0.43 | 0.16 |
| **35** | 0 | 0 | 0.2 | 0 | 0 | 0.2 | 0.3 | 0 | 0.1 | 0 | 0 | 0 | 0.2 | 0 | 128 | 0.43 | 0.50 |
| **36** | 0 | 0 | 0 | 0.3 | 0 | 0.3 | 0 | 0.1 | 0.1 | 0 | 0 | 0.1 | 0 | 0.1 | 126 | 0.43 | 0.83 |
| **37** | 0.2 | 0 | 0 | 0 | 0 | 0 | 0.2 | 0.1 | 0.3 | 0 | 0.1 | 0.1 | 0 | 0 | 128 | 0.50 | -0.84 |
| **38** | 0 | 0.2 | 0.1 | 0 | 0 | 0 | 0.2 | 0 | 0 | 0.3 | 0.2 | 0 | 0 | 0 | 126 | 0.50 | -0.50 |
| **39** | 0 | 0.1 | 0.1 | 0.2 | 0 | 0 | 0 | 0 | 0.4 | 0 | 0 | 0.2 | 0 | 0 | 126 | 0.50 | -0.17 |
| **40** | 0 | 0.1 | 0 | 0.1 | 0.1 | 0.1 | 0.1 | 0.2 | 0 | 0 | 0.1 | 0 | 0.2 | 0 | 134 | 0.50 | 0.17 |
| **41** | 0 | 0 | 0.1 | 0.2 | 0.1 | 0 | 0.3 | 0 | 0 | 0 | 0.1 | 0 | 0.2 | 0 | 128 | 0.50 | 0.50 |
| **42** | 0 | 0 | 0.1 | 0.1 | 0 | 0.3 | 0.1 | 0.2 | 0 | 0 | 0 | 0 | 0 | 0.2 | 132 | 0.50 | 0.84 |
| **43** | 0.2 | 0 | 0 | 0 | 0 | 0.2 | 0 | 0 | 0.1 | 0.2 | 0.3 | 0 | 0 | 0 | 132 | 0.56 | -0.83 |
| **44** | 0.1 | 0 | 0.2 | 0 | 0.1 | 0 | 0 | 0 | 0 | 0.5 | 0 | 0.1 | 0 | 0 | 128 | 0.56 | -0.50 |
| **45** | 0.1 | 0 | 0.1 | 0 | 0.2 | 0.1 | 0 | 0 | 0.1 | 0.1 | 0.2 | 0 | 0.1 | 0 | 128 | 0.56 | -0.17 |
| **46** | 0 | 0 | 0.2 | 0.2 | 0 | 0 | 0 | 0.2 | 0 | 0.2 | 0 | 0.1 | 0 | 0.1 | 132 | 0.56 | 0.17 |
| **47** | 0 | 0 | 0.1 | 0 | 0.5 | 0 | 0 | 0 | 0 | 0.1 | 0 | 0.2 | 0 | 0.1 | 132 | 0.56 | 0.50 |
| **48** | 0 | 0 | 0.1 | 0.2 | 0.1 | 0.1 | 0.2 | 0 | 0.1 | 0 | 0 | 0 | 0 | 0.2 | 126 | 0.56 | 0.84 |
| **49** | 0.1 | 0.2 | 0 | 0 | 0 | 0 | 0 | 0 | 0.1 | 0.4 | 0.2 | 0 | 0 | 0 | 132 | 0.62 | -0.83 |
| **50** | 0.1 | 0.2 | 0 | 0 | 0 | 0 | 0.1 | 0 | 0.3 | 0.1 | 0 | 0.2 | 0 | 0 | 126 | 0.62 | -0.50 |
| **51** | 0.1 | 0.1 | 0.1 | 0 | 0 | 0 | 0.1 | 0.2 | 0.1 | 0.1 | 0.1 | 0 | 0 | 0.1 | 126 | 0.62 | -0.16 |
| **52** | 0 | 0.1 | 0.1 | 0.2 | 0.1 | 0 | 0 | 0 | 0.1 | 0.2 | 0 | 0 | 0.2 | 0 | 126 | 0.62 | 0.17 |
| **53** | 0 | 0.1 | 0.1 | 0 | 0.1 | 0.4 | 0 | 0 | 0 | 0 | 0 | 0 | 0.3 | 0 | 126 | 0.62 | 0.50 |
| **54** | 0 | 0 | 0 | 0.2 | 0.4 | 0 | 0.1 | 0 | 0 | 0 | 0 | 0.1 | 0 | 0.2 | 130 | 0.62 | 0.81 |
| **55** | 0.2 | 0.1 | 0 | 0 | 0 | 0 | 0 | 0 | 0.1 | 0.3 | 0.3 | 0 | 0 | 0 | 132 | 0.69 | -0.82 |
| **56** | 0.1 | 0.2 | 0 | 0 | 0 | 0 | 0 | 0.2 | 0.1 | 0 | 0.3 | 0 | 0.1 | 0 | 132 | 0.69 | -0.50 |
| **57** | 0.2 | 0 | 0 | 0 | 0.2 | 0.1 | 0 | 0 | 0 | 0.3 | 0.1 | 0 | 0 | 0.1 | 126 | 0.69 | -0.16 |
| **58** | 0 | 0.1 | 0.1 | 0.2 | 0 | 0 | 0.2 | 0 | 0 | 0.1 | 0 | 0 | 0.3 | 0 | 132 | 0.69 | 0.17 |
| **59** | 0 | 0 | 0.1 | 0.2 | 0.3 | 0 | 0 | 0 | 0 | 0 | 0.2 | 0 | 0 | 0.2 | 132 | 0.69 | 0.50 |
| **60** | 0 | 0 | 0 | 0.3 | 0.3 | 0.1 | 0 | 0 | 0 | 0 | 0 | 0 | 0.1 | 0.2 | 128 | 0.69 | 0.82 |

**SUPPLEMENTARY TABLE 2**

**Response to Risk Dimensions:** A. Anatomical locations of regions positively correlating with the lottery variance on each trial. B. Anatomical locations of regions correlating with increasing positive skewness on each trial. C. Anatomical locations of regions correlating with increasing negative skewness on each trial. We report significant clusters surviving correction at p≤0.05 († = cluster-level family-wise error whole-brain corrected p-value), or significant voxels at p≤0.05 within regions of interest (†† = voxel-level family-wise error corrected p-value). Peak voxel MNI coordinates within significant clusters are given, with corresponding voxel-level Z scores. We define anatomical ROIs by 2cm-diameter spheres centred upon MNI coordinates for anterior insula/inferior frontal gyrus, ventral striatum, and anterior cingulate/dorso-medial prefrontal cortex, where risk-related activation has previously been reported.

| **Area** | **L/R** | **MNI coordinates** | | | **Z score** | **P value** | **Cluster Extent** |
| --- | --- | --- | --- | --- | --- | --- | --- |
|  |  | **x** | **y** | **z** |  |  |  |
| 1. **Response to Variance** | | | | | | | |
| Posterior Parietal Cortex | R | 32 | -60 | 50 | 3.71 | 0.003^†^ | 1318 |
|  |  | 28 | -46 | 46 | 3.66 |  |  |
|  |  | 16 | -62 | 48 | 3.59 |  |  |
| 1. **Response to Positive Skewness** | | | | | | | |
| Anterior insula / inferior frontal gyrus | R | 30 | 16 | -14 | 3.59 | 0.021^††^ | 117 |
| Anterior insula / inferior frontal gyrus | L | -40 | 24 | -16 | 3.59 | 0.017^††^ | 67 |
| Ventral Striatum | L | -10 | 4 | -14 | 3.36 | 0.033^††^ | 228 |
|  |  | -16 | 8 | -8 | 3.20 | 0.050^††^ |  |
| 1. **Response to Negative Skewness** | | | | | | | |
| Dorsal medial prefrontal cortex / medial frontal gyrus | R | 4 | 44 | 36 | 4.76 | <0.001^†^ | 1673 |
|  | L | -8 | 32 | 34 | 3.93 |  |  |
|  |  | -2 | 48 | 18 | 3.38 |  |  |

**SUPPLEMENTARY TABLE 3**

**Correlation of skew-related activity with skew-preference**: 2^nd^-level analysis of anatomical locations of regions where the strength of the skewness response correlated with subject-specific skew-preferences (estimated from **MVS** model). We restrict our analysis to, and perform family-wise error correction for multiple comparisons within all voxels sensitive to skewness (identified at p<0.01 uncorrected). Voxels reported at p<0.05 corrected.

| **Area** | **L/R** | **MNI coordinates** | | | **Z score** | **P value** | **Cluster Extent** |
| --- | --- | --- | --- | --- | --- | --- | --- |
|  |  | **x** | **y** | **z** |  |  |  |
| Anterior insula / inferior frontal gyrus | L | -36 | 24 | -16 | 4.36 | 0.007 | 80 |
| Anterior insula / inferior frontal gyrus / BA45 | L | -56 | 18 | 6 | 4.09 | 0.018 | 38 |

**SUPPLEMENTARY TABLE 4**

**Choice-related activity I**: BOLD signal correlating with choice within variance- and skew-sensitive regions of interest. We report significant voxels at p<0.05, family-wise error corrected for regions of interest.

| **Area** | **L/R** | **MNI coordinates** | | | **Z score** | **P value** | **Cluster Extent** |
| --- | --- | --- | --- | --- | --- | --- | --- |
|  |  | **x** | **y** | **z** |  |  |  |
| Posterior parietal cortex  (variance-sensitive voxels) | R | 26 | -60 | 54 | 4.03 | 0.008 | 547 |
|  |  | 30 | -68 | 30 | 3.97 | 0.010 |  |
|  |  | 18 | -62 | 30 | 3.70 | 0.023 |  |
| Ventral Striatum  (skew-sensitive voxels) | L | -8 | 4 | -10 | 3.11 | 0.016 | 39 |
|  |  | -16 | 4 | -15 | 2.83 | 0.033 |  |
| Medial Prefrontal cortex  (skew-sensitive voxels) | R | 6 | 44 | 18 | 3.53 | 0.049 | 525 |

**SUPPLEMENTARY TABLE 5**

**Choice-related activity II**: Anatomical locations of regions expressing greater BOLD signal for gamble versus sure choices. We report significant clusters surviving correction at p<0.05 (cluster-level family-wise error corrected). Peak voxel MNI coordinates within significant clusters are given, with corresponding voxel-level Z scores.

| **Area** | **L/R** | **MNI coordinates** | | | **Z score** | **P value** | **Cluster Extent** |
| --- | --- | --- | --- | --- | --- | --- | --- |
|  |  | **x** | **y** | **z** |  |  |  |
| Ventral Striatum | L | -6 | 8 | -4 | 4.59 | <0.001 | 2614 |
| Ventral Striatum | R | 18 | 22 | -2 | 4.40 |  |  |
| Middle Frontal Gyrus | R | 20 | 40 | -14 | 4.43 |  |  |
| Occipital Lobe/BA17 | L | -14 | -94 | -8 | 4.34 | <0.001 | 1742 |
| Occipital Lobe |  | -26 | -84 | -8 | 4.30 |  |  |
| Occipital Lobe/BA18 |  | -8 | -78 | -4 | 4.02 |  |  |
| Superior Parietal Lobe | L | -12 | -70 | 40 | 4.20 | 0.001 | 785 |
| Superior Parietal Lobe |  | -20 | -60 | 52 | 3.42 |  |  |
| Precuneus |  | -16 | -60 | 26 | 3.03 |  |  |
| Superior Parietal Lobe | R | 26 | -60 | 54 | 4.03 | <0.001 | 1248 |
| Parietal Lobe/Precuneus |  | 20 | -62 | 32 | 4.02 |  |  |
| Parietal Lobe/Mid-occipital gyrus |  | 30 | -68 | 30 | 3.97 |  |  |

**SUPPLEMENTARY TABLE 6**

**Interaction between choice and variance-preference**: Regions where neural response to choice (gamble>sure) correlated with individual variance-preference. We report significant clusters at p<0.05 family-wise error whole-brain corrected († - family-wise error small volume corrected for region showing interaction between skew preference and choice). Peak voxel MNI coordinates within significant clusters are given, with corresponding voxel-level Z scores.

| **Area** | **L/R** | **MNI coordinates** | | | **Z score** | **P value** | **Cluster Extent** |
| --- | --- | --- | --- | --- | --- | --- | --- |
|  |  | **x** | **y** | **z** |  |  |  |
| Superior Frontal Gyrus / Supplementary Motor Area | R | 6 | 20 | 58 | 4.10 | 0.014 | 790 |
|  |  | 6 | 34 | 38 | 3.28 |  |  |
|  |  | 8 | 24 | 36 | 3.73 |  |  |
| Posterior Cingulate / BA29 | L | -2 | -38 | 20 | 3.76 | 0.026 | 699 |
| Occipital Lobe / Lingual Gyrus | R | 20 | -72 | 0 | 3.43 |  |  |
| Occipital Lobe - Cuneus | R | 12 | -70 | 6 | 3.36 |  |  |
| Posterior Parietal Cortex / BA40 | R | 38 | -58 | 42 | 3.67 | 0.035 | 656 |
|  | R | 48 | -58 | 40 | 3.14 |  |  |
|  | R | 30 | -66 | 34 | 3.13 |  |  |
| Anterior insula / inferior frontal gyrus | R | 46 | -4 | -14 | 3.31 | 0.040^†^ | 57 |

**SUPPLEMENTARY TABLE 7**

**Interaction between choice and skew-preference**: Regions where neural response to choice (gamble>sure) correlated with individual skew preference. We report significant clusters at p<0.05 family-wise error corrected within regions of interest. Peak voxel MNI coordinates within significant clusters are given, with corresponding voxel-level Z scores.

| **Area** | **L/R** | **MNI coordinates** | | | **Z score** | **P value** | **Cluster Extent** |
| --- | --- | --- | --- | --- | --- | --- | --- |
|  |  | **x** | **y** | **z** |  |  |  |
| Anterior Insula / Inferior Frontal Gyrus | L | -24 | 22 | -6 | 3.78 | 0.033 | 666 |
|  |  | -10 | 12 | -12 | 3.29 |  |  |
|  |  | -38 | 4 | -4 | 3.11 |  |  |
| Insula / Superior Temporal Gyrus | R | 54 | -4 | -12 | 3.60 | 0.039 | 643 |
|  |  | 52 | 2 | -2 | 3.59 |  |  |
|  |  | 46 | -8 | -14 | 3.53 |  |  |
